# Modulatory role of endogenous adrenaline in propofol-related nociceptive responses in rats

**DOI:** 10.3389/fphar.2026.1773526

**Published:** 2026-03-20

**Authors:** Fusun Gozen, Esra Tuba Sezgin, Bulent Yavuzer, Cengiz Sarigul, Renad Mammadov, Bahadir Suleyman, Durdu Altuner, Halis Suleyman

**Affiliations:** 1 Department of Anesthesiology and Reanimation, Bursa Yuksek Ihtisas Training and Research Hospital, University of Health Sciences, Bursa, Türkiye; 2 Anesthesia Program, Vocational School of Health Services, Erzincan Binali Yıldırım University, Erzincan, Türkiye; 3 Department of Pharmacology, Faculty of Medicine, Erzincan Binali Yıldırım University, Erzincan, Türkiye; 4 Department of Medical Biochemistry, Faculty of Medicine, Erzincan Binali Yıldırım University, Erzincan, Türkiye

**Keywords:** adrenaline, anesthesia, mechanical paw withdrawal threshold, nociceptive suppression, propofol, rats

## Abstract

**Introduction:**

Propofol is a short-acting intravenous hypnotic anesthetic widely used for sedation and the induction and maintenance of general anesthesia. Although it reliably produces unconsciousness, its intrinsic analgesic efficacy remains a subject of debate. Accumulating evidence indicates that propofol suppresses catecholaminergic activity, and inhibition of endogenous adrenaline may therefore limit its ability to modulate nociceptive processing. Accordingly, this study aimed to investigate the nociceptive suppressions of subhypnotic and anesthetic doses of propofol in rats and their relationship with endogenous adrenaline levels.

**Methods:**

Thirty-six male Wistar rats were randomly allocated into six experimental groups: healthy healthy control, subhypnotic-dose propofol (25 mg/kg; PRO-25, intraperitoneally), anesthetic-dose propofol (50 mg/kg; PRO-50, intraperitoneally), adrenaline-alone (0.3 mg/kg; ADRG, intraperitoneally), and adrenaline combined with subhypnotic or anesthetic-dose propofol (PRAD-25 and PRAD-50). Mechanical paw withdrawal thresholds, anesthetic duration, behavioral responses to standardized scalpel incision, and plasma adrenaline levels were evaluated using statistical analyses.

**Results:**

Propofol administered alone failed to produce significant nociceptive suppressionat either dose and induced dose-dependent suppression of circulating adrenaline, whereas adrenaline alone exerted no significant effect on nociceptive thresholds. In contrast, combined administration significantly increased pain thresholds at subhypnotic doses and abolished responses to surgical incision at anesthetic doses despite comparable anesthetic duration. Notably, co-administration restored serum adrenaline levels toward physiological values at lower, but not higher, propofol doses.

**Conclusion:**

These findings provide experimental results suggesting that nociceptive responses observed during propofol exposure may be modulated by endogenous adrenergic activity. This interaction may contribute to explaining the dissociation between anesthetic depth and nociceptive suppression and points to a catecholaminergic influence on anesthetic–nociceptive dynamics that has not been sufficiently investigated previously.

## Graphical Abstract

**Figure:**
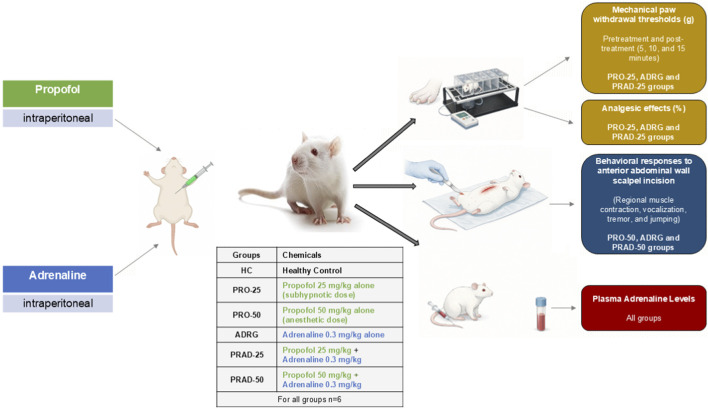

## 1 Introduction

Propofol is an intravenously administered, short-acting hypnotic anesthetic utilized for procedural sedation and for the induction and maintenance of general anesthesia (Dinis-Oliveira, 2018; Sahinovic et al., 2018). Its primary mechanism of action is based on facilitating the binding of γ-aminobutyric acid (GABA) to its receptor, thereby enhancing GABAergic activity (Levy, 2011; Sahinovic et al., 2018). Its rapid onset of action, swift systemic elimination, and low incidence of adverse effects, such as nausea, contribute to the preference for propofol as an intravenous anesthetic (Schulte et al., 2000; Tan et al., 2021). Following propofol administration, loss of consciousness typically occurs within approximately 100 s, and the anesthetic effect persists for 5–10 min (Levy, 2011). Although propofol is a preferred intravenous anesthetic, it induces vasodilation and causes a 25%–40% reduction in systolic and diastolic blood pressure (Levy, 2011; Beaty et al., 2022). In addition, bradycardia and hypotension are observed during propofol anesthesia (Kotani et al., 2008). Propofol-induced hypotension is considered to result from a reduction in plasma catecholamine levels. Lindgren et al. reported a reduction in plasma catecholamine levels during anesthesia induction with propofol (Lindgren et al., 1993). Propofol-induced inhibition of catecholamine release from adrenal medullary chromaffin cells and nerve terminals has been associated with reduced intracellular calcium (Ca^2+^) influx (Han et al., 2016). Although the literature suggests a potential analgesic role for propofol (Vasileiou et al., 2009), evidence supporting its nociceptive suppressions remains inconclusive. Teitelman stated that claims regarding the use of propofol for procedural sedation and analgesia are not convincing (Teitelman, 2014). Conversely, several studies indicate that propofol does not exert nociceptive suppressions (Bahrami Gorji et al., 2016). Previous studies have demonstrated that the nociceptive suppressions of intravenous anesthetic agents, such as ketamine and thiopental, are associated with adrenaline stimulation, whereas their hyperalgesic effects are linked to suppression of endogenous adrenaline production (Aksoy et al., 2014; Aksoy et al., 2015). Collectively, these findings suggest that the absence of analgesic activity observed with propofol may be attributable to its inhibitory effects on adrenaline production. However, no studies have been identified in the literature that specifically investigate the role of adrenaline in the potential nociceptive suppressions of propofol. Accordingly, the present study aimed to investigate the nociceptive suppressionsof subhypnotic and anesthetic doses of propofol in rats and to determine whether these effects are associated with endogenous adrenaline levels.

## 2 Materials and methods

### 2.1 Animals

This study was conducted using thirty-six male albino Wistar rats aged 9–10 weeks, with body weights ranging from 272 to 281 g. Animals used in this study were supplied by the Experimental Animals Application and Research Center of Erzincan Binali Yıldırım University (Erzincan, Türkiye). Animals were randomly allocated to six experimental groups (*n* = 6 per group), ensuring comparable baseline body weights among groups. Prior to the commencement of experimental procedures, the animals were allowed a 1-week acclimatization period and were maintained in standard wire cages (20 cm × 35 cm × 55 cm; floor area 1,925 cm^2^), with six rats housed per cage. Animals were maintained under controlled environmental conditions, including a 12 h light/12 h dark cycle, ambient temperature of 22 °C ± 2 °C, and relative humidity ranging from 30% to 70%. Animals were provided *ad libitum* access to standard laboratory chow (Bayramoglu Feed and Flour Industry Inc., Erzurum, Turkey) and tap water. All experimental interventions were carried out in the accredited laboratory units of the Experimental Animals Application and Research Center, Erzincan Binali Yıldırım University.

All experimental procedures were performed in compliance with Directive 2010/63/EU of the European Parliament for the protection of animals used for scientific purposes (Protocol ID: 2016-24-199). In addition, all experimental procedures and reporting were conducted in accordance with the ARRIVE 2.0 (Animal Research: Reporting of *In Vivo* Experiments) guidelines (Percie du Sert et al., 2020).

### 2.2 Reagents and chemicals

All chemicals and reagents used in the experimental protocols were of analytical grade and purchased from commercial suppliers. Thiopental sodium (Pental Sodyum®, 0.5 g vial; Catalog No.: 8699508270385) was supplied by Menarini Health and Pharmaceuticals Industry Trade Inc. (Istanbul, Turkey). Adrenaline (Adrenalin Biofarma®, 1 mg/mL ampoule; Catalog No.: 8699578750053) was obtained from Biofarma Pharmaceutical Industry and Trade Inc. (Istanbul, Turkey). Propofol (Propofol 1% Fresenius®, 200 mg/20 mL; Catalog No.: 8699630756009; Batch No.: 10MH6944) was provided by Fresenius Kabi Austria GmbH (Graz, Austria).

### 2.3 Experimental design and randomization

The sample size was determined in accordance with the principle of minimizing animal use while ensuring the generation of robust and reproducible results, consistent with the 4R framework (Reduction, Refinement, Replacement, and Responsibility) (Kiani et al., 2022). Two sequential phases of exclusion criteria were incorporated into the experimental design. During the pre-experimental phase, animals exhibiting abnormal posture, reduced spontaneous locomotor activity, or injuries resulting from inter-animal aggression were excluded prior to randomization and initiation of the experimental intervention. Peri- and post-experimental exclusion criteria comprised events such as unexpected mortality or complications related to anesthesia or drug administration prior to the planned endpoints; procedural errors during dosing, including extravasation during injection; deviations from the intended treatment schedule or incomplete administration of study compounds; body weight loss exceeding 15%–20% of baseline, dehydration, or clinical signs of systemic illness; severe distress, self-injurious behavior, or persistent vocalization indicative of uncontrolled pain or suffering; inability to complete behavioral assessments due to non-compliance or motor impairments unrelated to the experimental interventions; and loss of tissue integrity during collection or processing, precluding reliable histological or biochemical analyses. Application of the exclusion criteria was maintained throughout the intervention period and subsequent data evaluation. None of the animals met the predefined exclusion thresholds during the pre-experimental or peri- and post-experimental phases; therefore, no animals were excluded from the study. Animals were allocated to experimental groups using a random number table. To minimize potential confounding variables and systematic bias, unique numerical identification codes were assigned to cages and individual animals and retained throughout the study.

### 2.4 Experimental groups

Following random allocation, animals were distributed into six experimental groups: HC (healthy control); PRO-25, receiving propofol alone at 25 mg/kg intraperitoneally (i.p.); PRO-50, receiving propofol alone at 50 mg/kg i.p.; ADRG, receiving adrenaline alone at 0.3 mg/kg i.p.; PRAD-25, receiving adrenaline (0.3 mg/kg, i.p.) in combination with propofol (25 mg/kg, i.p.); and PRAD-50, receiving adrenaline (0.3 mg/kg, i.p.) in combination with propofol (50 mg/kg, i.p.).

### 2.5 Experimental procedure

Animals in the PRO-25 group (*n* = 6) were injected i.p. with propofol at a subhypnotic dose (25 mg/kg) (Liu et al., 2016), whereas animals in the PRO-50 group (*n* = 6) were injected i.p. with propofol at an anesthetic dose (50 mg/kg) (Pesic et al., 2009). Animals in the healthy control group (*n* = 6) received distilled water i.p. as the vehicle. Adrenaline (0.3 mg/kg, i.p.) was administered to animals in the ADRG (*n* = 6), PRAD-25 (*n* = 6), and PRAD-50 (*n* = 6) groups (Aksoy et al., 2015). Following adrenaline administration, animals in the PRAD-25 and PRAD-50 groups were subsequently injected with propofol i.p. at doses of 25 mg/kg and 50 mg/kg, respectively. Paw withdrawal thresholds in the PRO-25 and PRAD-25 groups were assessed using a Basile analgesimeter (Randall–Selitto method) (Cadirci et al., 2010), before propofol administration and at 5, 10, and 15 min thereafter. In addition, paw withdrawal thresholds in the ADRG group were measured before adrenaline administration and at 5, 10, and 15 min following administration. All measurements were conducted according to predefined standardized criteria by a single investigator who was blinded to the treatment group allocation. In the PRO-50, ADRG and PRAD-50 groups, both the duration of anesthesia and nociceptive responses of the animals were assessed. The duration during which animals remained motionless in the supine position was defined as a surgically adequate duration of anesthesia (Kurt et al., 2011). While the animals remained immobile in the supine position, nociceptive responses were assessed using a standardized 1-cm vertical abdominal incision (scalpel incision). Serum adrenaline concentrations were determined in blood samples collected from the tail vein 10 min after propofol administration. At the end of the experimental protocol, all animals were euthanized by an overdose of thiopental sodium (50 mg/kg, i.p.), administered to induce deep surgical anesthesia resulting in cessation of cardiac activity and respiration, in accordance with institutional and international guidelines for animal care. Data obtained from all experimental groups were subsequently subjected to comparative statistical analysis.

#### 2.5.1 Assessment of paw withdrawal threshold

Mechanical nociceptive sensitivity was assessed using the paw withdrawal threshold test. The force at which the animal withdrew its paw was recorded as the withdrawal threshold. Measurements were obtained prior to drug administration (baseline value) and at the designated post-treatment time points. The nociceptive suppression was expressed as the percentage change relative to the baseline threshold and was calculated individually for each animal using the following formula:

% Analgesic Effect = [(Post − treatment threshold − Baseline threshold) / Baseline threshold] × 100. Group data were presented as mean ± SEM (standard error of the mean) of the individual percentage changes.

### 2.6 Biochemical analyses

#### 2.6.1 Determination of plasma ADR levels in rats

To determine plasma adrenaline concentrations, blood samples (2 mL) were collected from the tail vein of rats into EDTA-containing vacuum tubes and immediately placed on ice. Samples were centrifuged at 4,100 rpm for 15 min at 4 °C. Following centrifugation, plasma was carefully separated and stored at −80 °C until analysis. Plasma adrenaline concentrations were quantified using an Agilent 6460 Triple Quadrupole Liquid Chromatography–Tandem Mass Spectrometry (LC–MS/MS) system (Santa Clara, CA, United States) in accordance with standard analytical procedures.

### 2.7 Statistical analysis

All statistical analyses of mechanical paw withdrawal thresholds and biochemical parameters were conducted using IBM SPSS® Statistics for Windows, version 27.0 (IBM Corp., Armonk, NY, United States; 2020). Figures and graphical representations were generated using GraphPad Prism® software (version 8.0.1; GraphPad Software, San Diego, CA, United States; 2018). Mechanical paw withdrawal thresholds (g) are presented as mean ± standard error of the mean (SEM). Normality of the mechanical paw withdrawal threshold data was assessed using the Shapiro–Wilk test (Supplementary Table S1). Within-group time-dependent changes at baseline and at 5, 10, and 15 min post-treatment were analyzed using repeated measures analysis of variance (RM-ANOVA). The assumption of sphericity was evaluated using Mauchly’s test; when sphericity was satisfied, sphericity-assumed results were reported, whereas appropriate corrections were applied when this assumption was violated. Post hoc pairwise comparisons between time points were conducted using Bonferroni adjustment for multiple comparisons. Biochemical outcomes are presented as mean values together with the corresponding SEM. Normality of biochemical data distribution was evaluated using the Shapiro–Wilk test (Supplementary Table S2), whereas homogeneity of variances was assessed using Levene’s test (Supplementary Table S3). For intergroup comparisons, one-way analysis of variance (ANOVA) was performed, followed by Tukey’s honestly significant difference (HSD) *post hoc* test when the assumption of variance homogeneity was satisfied; when this assumption was not met, Welch’s ANOVA followed by the Games–Howell *post hoc* test was applied. Statistical significance was defined as *p* < 0.05.

## 3 Results

### 3.1 Results of pain-related behavioral tests

#### 3.1.1 Within-group time-dependent effects of propofol, adrenaline, and propofol–adrenaline combination

**FIGURE 1:**
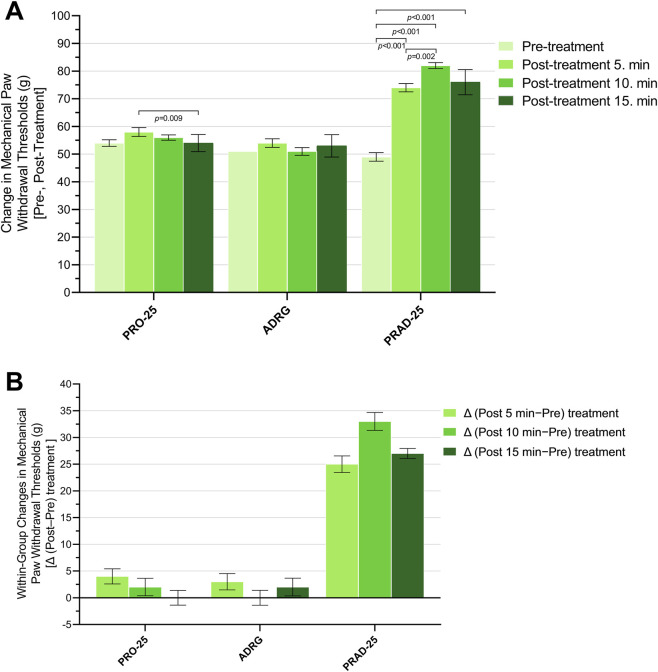
**(A)** Mechanical paw withdrawal thresholds (g) assessed at baseline (pre-treatment) and at 5, 10, and 15 min after treatment, and **(B)** corresponding within-group changes in mechanical paw withdrawal thresholds, expressed as Δ (post-treatment minus pre-treatment), at 5, 10, and 15 min, in the PRO-25, ADRG, and PRAD-25 groups. Data are expressed as mean ± SEM (*n* = 6 per group). Within-group comparisons were evaluated using repeated measures analysis of variance (RM-ANOVA). As the assumption of sphericity was met, sphericity-assumed results were reported. Bonferroni-adjusted *post hoc* tests were applied for pairwise comparisons. Only statistically significant differences are presented in the figure. Abbreviations: PRO-25, propofol alone (25 mg/kg); ADRG, adrenaline alone (0.3 mg/kg); PRAD-25, adrenaline (0.3 mg/kg) combined with propofol (25 mg/kg); Δ, post-treatment minus pre-treatment; min, minute(s).

The within-group time-dependent effects of propofol, adrenaline, and their combination on pain-related behavioral outcomes are presented in Figures 1A,B and Tables 1 and 2 and Supplementary Table S4. For the PRO-25 group, Mauchly’s test indicated that the assumption of sphericity was not violated (W = 0.234, χ^2^ (5) = 5.400, *p* = 0.383); therefore, sphericity-assumed results are reported. Repeated measures ANOVA demonstrated a statistically significant main effect of time in the PRO-25 group (F (3, 15) = 5.08, *p* = 0.013, partial η^2^ = 0.504) (Figure 1A). Nevertheless, Bonferroni-adjusted *post hoc* analyses revealed a significant difference exclusively between the 5th and 15th minutes (*p* = 0.009), whereas all other pairwise comparisons were not statistically significant (p > 0.05). These findings indicate that PRO-25 produced only a weak and transient nociceptive suppression on mechanical paw withdrawal threshold (Figure 1A), emerging within the early post-administration period (5–15 min) and not sustained thereafter.

**TABLE 1:** Effects of a subhypnotic dose of propofol (25 mg/kg) on mechanical paw withdrawal threshold. A. Within-group changes in mechanical paw withdrawal threshold over time and repeated measures ANOVA results.

Groups	Mechanical paw withdrawal threshold (g)							​		
	​	Post-treatment			Δ (post−pre) treatment			​		
	Baseline	5 min	10 min	15 min	5 min	10 min	15 min	F (df1, df2)	*p* (time effect)	η^2^p
PRO-25	54 ± 1.21	58 ± 1.59	56 ± 0.97	54 ± 1.26	4 ± 1.41	2 ± 1.63	0 ± 1.37	F (3, 15) = 5.08	0.013	0.504
ADRG	51 ± 0.00	54 ± 1.53	51 ± 1.39	53 ± 1.65	3 ± 1.53	0 ± 1.39	2 ± 1.65	F (3, 15) = 2.49	0.100	0.332
PRAD-25	49 ± 1.53	74 ± 1.48	82 ± 1.06	76 ± 1.86	25 ± 1.55	33 ± 1.67	27 ± 0.93	F (3, 15) = 198.98	<0.001	0.975

Data are presented as mean ± SEM (standard error of the mean). Δ indicates the change calculated as post–pre.

Abbreviations: PRO-25, propofol alone (25 mg/kg); ADRG, adrenaline alone (0.3 mg/kg); PRAD-25, adrenaline (0.3 mg/kg) combined with propofol (25 mg/kg); Baseline, pretreatment; min, minute(s); F, F statistic for the within-subjects main effect of time (repeated measures ANOVA); df1, numerator degrees of freedom (time); df2, denominator degrees of freedom (error); η^2^p, partial eta squared.

**TABLE 2:** Effects of a subhypnotic dose of propofol (25 mg/kg) on mechanical paw withdrawal threshold. B. Within-group pairwise comparisons.

Groups	Bonferroni-adjusted *p* values					
	Baseline vs. 5 min	Baseline vs. 10 min	Baseline vs. 15 min	5 vs. 10 min	5 vs. 15 min	10 vs. 15 min
PRO-25	0.220	1.000	1.000	0.765	0.009	0.245
ADRG	0.640	1.000	1.000	0.202	1.000	0.957
PRAD-25	<0.001	<0.001	<0.001	0.002	1.000	0.103

Pairwise comparisons were performed using Bonferroni adjustment for multiple comparisons. Values represent adjusted p values. Statistically significant differences are indicated in bold (*p* < 0.05).

PRO-25, propofol alone (25 mg/kg); ADRG, adrenaline alone (0.3 mg/kg); PRAD-25, adrenaline (0.3 mg/kg) combined with propofol (25 mg/kg); Baseline, pretreatment; min, minute(s).

For the ADRG group, Mauchly’s test indicated that the assumption of sphericity was not violated (W = 0.734, χ^2^ (5) = 1.151, *p* = 0.951); therefore, sphericity-assumed results are reported. In contrast to the PRO-25 group, repeated measures ANOVA revealed no significant main effect of time on the outcome measure in the ADRG group (F (3, 15) = 2.49, *p* = 0.100, partial η^2^ = 0.332). Consistent with the absence of an overall time effect, Bonferroni-adjusted *post hoc* pairwise comparisons demonstrated no significant differences between any of the evaluated time points (all p > 0.05). These results demonstrate that adrenaline administered alone did not significantly alter mechanical paw withdrawal thresholds over time (Figure 1A), indicating the absence of a meaningful nociceptive suppression under the present experimental conditions.

For the PRAD-25 group, Mauchly’s test indicated that the assumption of sphericity was not violated (W = 0.334, χ^2^ (5) = 4.085, *p* = 0.549); therefore, sphericity-assumed results are reported. In this group, repeated measures ANOVA revealed a highly significant and robust main effect of time on the outcome measure (F (3, 15) = 198.98, *p* < 0.001), with a very large effect size (partial η^2^ = 0.975). Bonferroni-adjusted *post hoc* pairwise comparisons revealed significant differences between baseline and all post-treatment time points (5, 10, and 15 min; all *p* < 0.001), as well as a significant difference between the fifth and 10th minutes (*p* = 0.002). By contrast, no significant differences were observed between the 5th and 15th minutes or between the 10th and 15th minutes (p > 0.05). Collectively, these findings indicate that combined administration of adrenaline and subhypnotic-dose propofol produced a robust suppression of nociceptive responses, characterized by a marked increase in paw withdrawal threshold beginning at 5 min and persisting throughout the observation period (Figure 1A).

#### 3.1.2 Nociceptive suppression associated with propofol, adrenaline, and their combination

As shown in Table 3, administration of 25 mg/kg propofol alone (PRO-25) failed to elicit a meaningful nociceptive suppression at any of the evaluated time points, with analgesia rates of 7% at 5 min, 3.57% at 10 min, and 0% at 15 min. Similarly, animals treated with adrenaline alone (ADRG) did not exhibit notable analgesia, as evidenced by values of 6% at 5 min, 0% at 10 min, and 4% at 15 min. In contrast, the combined administration of adrenaline and 25 mg/kg propofol (PRAD-25) elicited a clear analgesic response, with analgesia rates of 34% at 5 min, 40% at 10 min, and 36% at 15 min. Importantly, no anesthetic effect was observed in any experimental group at the assessed time points. Taken together, these percentage analyses confirm that neither propofol nor adrenaline alone produced biologically meaningful change in nociceptive threshold, whereas their combined administration resulted in a pronounced and consistent nociceptive suppression across all evaluated time points.

**TABLE 3:** Analgesic effects of treatments administered at subhypnotic dose level (mechanical paw withdrawal threshold analysis).

Groups	Post-treatment analgesic effects (%)		
	5 min	10 min	15 min
PRO-25	7	4	0.00
ADRG	6	0.00	3.8
PRAD-25	34	40	36

No anesthetic effect was observed in any experimental group at the assessed time points. Values are expressed as percentages. For all groups, *n* = 6.

Abbreviations: min, minute(s); PRO-25, propofol alone (25 mg/kg); ADRG, adrenaline alone (0.3 mg/kg); PRAD-25, adrenaline (0.3 mg/kg) combined with propofol (25 mg/kg).

### 3.2 Analgesic and anesthetic effects of propofol, adrenaline, and their combination

As shown in Table 4, administration of 50 mg/kg propofol alone (PRO-50) induced anesthesia in all animals, with a mean anesthetic duration of 7.50 ± 0.17 min. However, despite the presence of anesthesia, animals exhibited behavioral responses to scalpel incision applied to the anterior abdominal wall, including regional muscle contraction, vocalization, tremor, and jumping. A similar pattern of nociceptive responsiveness was observed in animals treated with adrenaline alone (ADRG); notably, adrenaline alone did not induce anesthesia in any animal.

**TABLE 4:** Behavioral responses of rats to scalpel incision at the anesthetic dose of propofol.

Groups	Duration of anesthesia (min)	Behavioral response to scalpel incision
PRO-50	7.50 ± 0.17	+
ADRG	−	+
PRAD-50	6.85 ± 0.16	−

Responses to scalpel incision were evaluated based on the presence of regional muscle contraction, vocalization, tremor, and jumping. (+) indicates the presence of a behavioral response to scalpel incision; (−) indicates the absence of a behavioral response to scalpel incision. For all groups, *n* = 6.

Abbreviations: min, minute(s); PRO-50, propofol alone (50 mg/kg); ADRG, adrenaline alone (0.3 mg/kg); PRAD-50, adrenaline (0.3 mg/kg) combined with propofol (50 mg/kg); min, minute(s).

In contrast, animals receiving the combined administration of adrenaline and 50 mg/kg propofol (PRAD-50) developed anesthesia with a mean duration of 6.85 ± 0.16 min, and no behavioral response to scalpel incision was observed in this group, demonstrating effective suppression of both nociceptive and motor reactions under combined treatment. These findings indicate that propofol-induced anesthesia alone was insufficient to suppress nociceptive responses to surgical stimulation, whereas the addition of adrenaline effectively abolished both motor and behavioral reactions, demonstrating enhanced suppression of nociceptive and motor responses under combined treatment conditions.

#### 3.2.1 Effect of subhypnotic and anesthetic doses of propofol on serum adrenaline levels

**FIGURE 2:**
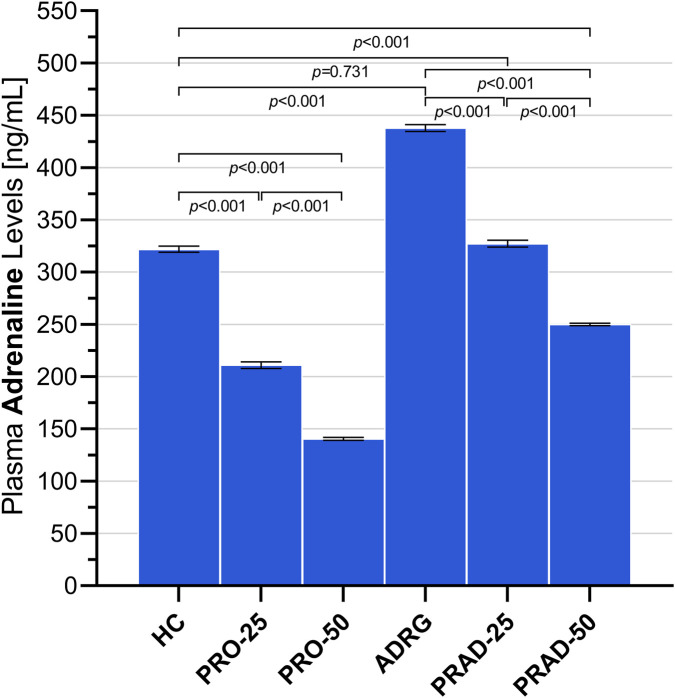
Effects of subhypnotic and anesthetic doses of propofol, administered alone or in combination with adrenaline, on plasma adrenaline levels in rats. Data are expressed as mean ± SEM (*n* = 6 per group). Intergroup differences were evaluated using one-way analysis of variance (ANOVA), followed by Tukey’s honestly significant difference (HSD) *post hoc* test. Abbreviations: HC, healthy control; PRO-25, propofol alone (25 mg/kg); PRO-50, propofol alone (50 mg/kg); ADRG, adrenaline alone (0.3 mg/kg); PRAD-25, adrenaline (0.3 mg/kg) combined with propofol (25 mg/kg); PRAD-50, adrenaline (0.3 mg/kg) combined with propofol (50 mg/kg).

As illustrated in Figure 2, propofol administration induced a significant and dose-dependent reduction in serum ADR levels. Treatment with 25 mg/kg propofol (PRO-25, 211.00 ± 3.13) significantly reduced ADR levels compared with the healthy control group (HC, 321.83 ± 3.03), with the difference reaching statistical significance (PRO-25 vs. HC, *p* < 0.01). A more pronounced suppression of ADR levels was observed following administration of 50 mg/kg propofol (PRO-50, 140.50 ± 1.38), leading to a highly significant reduction compared with HC (PRO-50 vs. HC, *p* < 0.001). In addition, ADR levels differed significantly between the two propofol monotherapy groups, confirming a clear dose-dependent effect (PRO-25 vs. PRO-50, *p* < 0.001).

Animals receiving adrenaline alone (ADRG, 437.83 ± 3.28) exhibited markedly elevated serum ADR levels relative to HC (ADRG vs. HC, *p* < 0.001). In contrast, co-administration of adrenaline with 25 mg/kg propofol (PRAD-25, 327.17 ± 3.32) restored ADR levels to values comparable to those of the healthy controls, with no statistically significant difference observed (PRAD-25 vs. HC, *p* = 0.731). However, in the PRAD-50 group (249.83 ± 1.17), ADR levels remained significantly lower than those in HC (PRAD-50 vs. HC, *p* < 0.001), demonstrating that higher-dose propofol preserved its suppressive effect on circulating adrenaline even in the presence of exogenous ADR administration. Overall, these biochemical findings support a dose-dependent suppressive effect of propofol on endogenous adrenaline and suggest that restoration of adrenergic activity may contribute to modulation of propofol-related nociceptive processing observed under combined treatment conditions.

## 4 Discussion

The present study examined the nociceptive responses associated with propofol administrationat subhypnotic and anesthetic doses in rats and explored the relationship between these effects and endogenous adrenaline levels. In addition, the dose-dependent effects of propofol on adrenaline levels were evaluated, and the association between these alterations and the observed analgesic responses was examined.

Propofol is primarily recognized as an intravenous anesthetic agent characterized by a rapid onset and short duration of action; however, whether it possesses intrinsic analgesic properties remains a matter of debate in the literature (Vasileiou et al., 2009; Teitelman, 2014). Evidence from prior studies indicates that intravenous anesthetics, including ketamine and thiopental, exert nociceptive suppressionsvia stimulation of adrenaline release, whereas suppression of endogenous adrenaline production has been implicated in their hyperalgesic actions (Aksoy et al., 2014; Aksoy et al., 2015). Our findings indicate that propofol administered at 25 mg/kg does not exert a statistically significant analg nociceptive suppressionesic effect. Petersen-Felix et al. similarly demonstrated that propofol, when administered at subhypnotic doses, fails to produce nociceptive suppressionsagainst a range of experimental stimuli (Petersen-Felix et al., 1996). By contrast, although several studies suggest that elevations in adrenaline levels may enhance analgesia (Aksoy et al., 2014; Aksoy et al., 2015), this view is not consistently supported across the literature. Indeed, other evidence indicates that adrenaline may exert a bidirectional modulatory influence on analgesic responses, thereby possessing the capacity to both enhance and attenuate analgesia (Wyns et al., 2023). For example, the weak and short-lasting nociceptive suppression of meperidine has been reported to result from its ability to elevate serum adrenaline levels (Yilmaz et al., 2023). While neither propofol administered alone at a subhypnotic dose nor adrenaline administered alone produced a significant nociceptive suppression, co-administration of the two agents resulted in a significant increase in the nociceptive threshold. Although several comparisons reached statistical significance, not all findings corresponded to biologically meaningful effects. In particular, the modest threshold variations observed with propofol monotherapy remained clearly limited when contrasted with the very large effect size detected in the combined treatment group. This underscores the importance of interpreting p values together with effect magnitude. Consistent with the findings reported by Hempel et al., our results indicate a significant reduction in adrenaline levels following propofol administration (Hempel et al., 2020). In our study, the presence of a behavioral response to scalpel incision in the group receiving propofol at 50 mg/kg may be interpreted as indicative of inadequate pain control. Fassoulaki similarly suggested that propofol cannot be regarded as a potent analgesic agent (Fassoulaki, 2011). Our findings indicate that, in the adrenaline-alone-treated group, the incision was likewise perceived as a painful nociceptive stimulus. Janssen et al. reported that adrenaline infusion was associated with increased subjective pain scores and reduced heat pain thresholds (Janssen et al., 1998). Consistent with the existing literature, our data further strengthen the evidence that adrenaline alone does not exert a pronounced nociceptive suppression. However, no behavioral response to scalpel incision was observed following the combined administration of propofol at 50 mg/kg and adrenaline. These data indicate that there may be a modulatory interaction between propofol administration and endogenous catecholaminergic activity in shaping nociceptive response. Alternative physiological explanations should also be considered. Hemodynamic alterations, stress-axis activation, and systemic catecholamine redistribution may contribute to the observed interaction between propofol and adrenaline, independent of direct nociceptive pathway modulation. When anesthesia duration was evaluated, the 7.5-min duration observed with the anesthetic dose of propofol was reduced in the PRAD-50 group, although this reduction did not reach statistical significance. Although adrenaline is well known to prolong anesthetic duration when co-administered with local anesthetics (Tschopp et al., 2018; Bajwa et al., 2023), there is insufficient evidence in the literature to suggest that it alters the duration of anesthesia under general anesthesia. It has been demonstrated that, under conditions of suppressed endogenous adrenaline, anesthesia can be achieved even at subhypnotic doses of ketamine (Aksoy et al., 2014). Another study demonstrated that thiopental induces hyperalgesia in rats by reducing adrenaline production and further indicated that, under general anesthesia, co-administration with adrenaline is required to elicit its analgesic activity (Aksoy et al., 2015). Several studies in the literature have demonstrated the suppressive effects of propofol on catecholaminergic responses, and our findings are consistent with these reports. Engelhard et al. reported that, in an experimental model of cerebral ischemia, propofol markedly inhibited catecholaminergic responses in a dose-dependent manner (Engelhard et al., 2003). Similarly, Minami et al. demonstrated that propofol dose-dependently reduced catecholamine secretion and reuptake in cultured adrenal medullary cells (Minami et al., 1996). At the clinical level, Jung and Cho demonstrated that deep propofol anesthesia significantly reduced stress hormone levels as well as circulating adrenaline concentrations in patients undergoing open lung surgery (Jung and Cho, 2015). When viewed as a whole, these studies provide strong evidence that propofol suppresses the catecholaminergic response, and our findings regarding adrenaline levels appear to be consistent with this mechanism.

## 5 Conclusion

This study investigated the nociceptive responsesof propofol administered at subhypnotic and anesthetic doses and explored the relationship between these effects and endogenous adrenaline levels. Our findings demonstrate that neither propofol nor adrenaline administered alone produced a significant suppression of nociception. Propofol was found to markedly suppress serum adrenaline levels in a dose-dependent manner, a mechanism that may partially account for the absence of nociceptive attenuationwhen propofol is used as a single agent. In contrast, the combined administration of propofol and adrenaline elicited a pronounced analgesic response at both subhypnotic and anesthetic doses. The magnitude of this effect appears to exceed what could be explained solely by modulation of catecholaminergic activity, suggesting that the concomitant use of these agents may engage additional interactive mechanisms involved in pain modulation.

With respect to anesthesia duration, although the propofol–adrenaline combination did not significantly alter anesthetic depth, the complete abolition of motor responses to scalpel incision provides compelling evidence that the combined treatment substantially enhances analgesic efficacy.

Taken together, our study addresses an important gap in the literature by demonstrating that adrenaline may play a modulatory role in the modulatory influenceof propofol. These findings underscore the importance of considering both endogenous and exogenous catecholamine dynamics when evaluating nociceptive responses associated with propofol and may provide a framework for future experimental and translational investigations.

### 5.1 Limitations

This study has several limitations that should be acknowledged. First, the investigation was conducted using a limited range of propofol doses and a single adrenaline dose, which constrains evaluation of dose–response relationships and the full dynamic spectrum of adrenergic modulation. Second, the absence of a positive control group receiving a well-established analgesic agent limits direct benchmarking of the observed effects against a recognized reference standard for nociceptive suppression. Third, the exclusive use of male rats precludes assessment of sex-specific differences in nociceptive processing, adrenergic signaling, and anesthetic–nociceptive interactions, thereby limiting generalizability across sexes. Fourth, although the rat model reliably captures fundamental aspects of nociceptive processing and adrenergic signaling, species-specific differences in pain perception, anesthetic sensitivity, and catecholamine regulation restrict direct extrapolation to human clinical settings. Fifth, endogenous adrenaline was assessed at predefined time points rather than through continuous temporal profiling, potentially limiting resolution of rapid catecholaminergic fluctuations following drug administration. Sixth, behavioral nociceptive outcomes, despite standardized and blinded assessment, inherently exhibit biological variability, introducing a degree of imprecision. Seventh, downstream molecular signaling pathways of adrenergic receptor activation and other neuromodulators influencing propofol-related analgesia (e.g., cortisol, GABA, serotonin, dopamine) were not evaluated. An additional limitation is the intraperitoneal administration of propofol. Considering that propofol is used clinically *via* the intravenous route, it is conceivable that intravenous administration might have resulted in more pronounced analgesic and anesthetic effects. Finally, the relatively small sample size represents a limitation and may restrict the generalizability of the findings.

Collectively, these limitations underscore the need for future studies incorporating broader dose ranges, positive analgesic controls, inclusion of both sexes, extended temporal analyses, receptor-specific and molecular approaches, and multi-neuromediator profiling to further refine mechanistic understanding and translational relevance.

## Data Availability

The original contributions presented in the study are included in the article/Supplementary Material, further inquiries can be directed to the corresponding author.
