# Supplementary material for: Modulatory role of endogenous adrenaline in propofol-related nociceptive responses in rats
Source: Front Pharmacol. 2026 Mar 20;17:1773526. doi: 10.3389/fphar.2026.1773526 (PMC13047178; doi:10.3389/fphar.2026.1773526)
Supplement: Supplementary file 1 [file Table1.docx]

**Supplementary Table S1.** Normality assessment of mechanical paw withdrawal thresholds in rats treated with subhypnotic doses of propofol, administered alone or in combination with adrenaline, using the Shapiro–Wilk test.

|  |  |  | **Groups** |  |
| --- | --- | --- | --- | --- |
|  | **Shapiro-Wilk** | **PRO-25** | **ADRG** | **PRAD-25** |
| Baseline | Statistic | 0.790 | - | 0.937 |
|  | df | 6 | 6 | 6 |
|  | Sig. | 0.048 | - | 0.634 |
| Post-treatment 5 min | Statistic | 0.943 | 0.898 | 0.867 |
|  | df | 6 | 6 | 6 |
|  | Sig. | 0.686 | 0.362 | 0.216 |
| Post-treatment 10 min | Statistic | 0.950 | 0.889 | 0.960 |
|  | df | 6 | 6 | 6 |
|  | Sig. | 0.739 | 0.313 | 0.817 |
| Post-treatment 15 min | Statistic | 0.867 | 0.913 | 0.880 |
|  | df | 6 | 6 | 6 |
|  | Sig. | 0.213 | 0.457 | 0.270 |

**Footnotes:** As most datasets satisfied the assumption of normality, ANOVA was applied for intergroup parametric statistical analyses, which are considered appropriate and robust for small sample sizes. For all groups, *n* = 6.

**Abbreviations**: PRO-25, propofol alone (25 mg/kg); ADRG, adrenaline alone (0.3 mg/kg); PRAD-25, adrenaline (0.3 mg/kg) combined with propofol (25 mg/kg); min, minute(s); df, degrees of freedom; Sig, significance; Baseline, pre-treatment.
