# Supplementary material for: Modulatory role of endogenous adrenaline in propofol-related nociceptive responses in rats
Source: Front Pharmacol. 2026 Mar 20;17:1773526. doi: 10.3389/fphar.2026.1773526 (PMC13047178; doi:10.3389/fphar.2026.1773526)
Supplement: Supplementary file 2 [file Table2.docx]

**Supplementary Table S2.** Assessment of normality for plasma adrenaline levels in rats using the Shapiro–Wilk test

|  |  |  | **Groups** | | | | | |
| --- | --- | --- | --- | --- | --- | --- | --- | --- |
|  |  | **Shapiro-Wilk** | **HC** | **PRO-25** | **PRO-50** | **ADRG** | **PRAD-25** | **PRAD-50** |
| Biochemical Variable | Adrenaline | Statistic | 0.925 | 0.978 | 0.967 | 0.979 | 0.880 | 0.989 |
|  |  | df | 6 | 6 | 6 | 6 | 6 | 6 |
|  |  | Sig. | 0.542 | 0.943 | 0.869 | 0.947 | 0.268 | 0.987 |

**Footnotes:** As plasma adrenaline datasets satisfied the assumption of normality, intergroup comparisons were performed using ANOVA. For all groups, *n* = 6.

**Abbreviations**: PRO-25, propofol alone (25 mg/kg); PRO-50, propofol alone (50 mg/kg); ADRG, adrenaline alone (0.3 mg/kg); PRAD-25, adrenaline (0.3 mg/kg) combined with propofol (25 mg/kg); PRAD-50, adrenaline (0.3 mg/kg) combined with propofol (50 mg/kg); df, degrees of freedom; Sig, significance.
