# Supplementary material for: Modulatory role of endogenous adrenaline in propofol-related nociceptive responses in rats
Source: Front Pharmacol. 2026 Mar 20;17:1773526. doi: 10.3389/fphar.2026.1773526 (PMC13047178; doi:10.3389/fphar.2026.1773526)
Supplement: Supplementary file 3 [file Table3.docx]

**Supplementary Table S3.** Assessment of variance homogeneity for plasma adrenaline levels

|  |  | **Levene’s statistic** | **df1** | **df2** | **Sig.** |
| --- | --- | --- | --- | --- | --- |
| Biochemical Variable | Adrenaline | 1.949 | 5 | 30 | 0.116 |

**Footnotes:** As the assumption of homogeneity of variances was satisfied, Tukey’s Honestly Significant Difference (HSD) test was applied for post hoc comparisons of plasma adrenaline levels. For all groups, *n* = 6.

**Abbreviations**: df, degrees of freedom; Sig, significance.
