# Supplementary material for: Modulatory role of endogenous adrenaline in propofol-related nociceptive responses in rats
Source: Front Pharmacol. 2026 Mar 20;17:1773526. doi: 10.3389/fphar.2026.1773526 (PMC13047178; doi:10.3389/fphar.2026.1773526)
Supplement: Supplementary file 4 [file Table4.docx]

**Supplementary Table S4.** Comparative effects of adrenaline and propofol on plasma adrenaline levels in rats

|  | **Post hoc**  ***p*-values** |
| --- | --- |
| **Group comparisons** | **Adrenaline** |
| HC vs. PRO-25 | <0.001 |
| HC vs. PRO-50 | <0.001 |
| HC vs. ADRG | <0.001 |
| HC vs. PRAD-25 | 0.731 |
| HC vs. PRAD-50 | <0.001 |
| PRO-25 vs. PRO-50 | <0.001 |
| PRO-25 vs. ADRG | <0.001 |
| PRO-25 vs. PRAD-25 | <0.001 |
| PRO-25 vs. PRAD-50 | <0.001 |
| PRO-50 vs. ADRG | <0.001 |
| PRO-50 vs. PRAD-25 | <0.001 |
| PRO-50 vs. PRAD-50 | <0.001 |
| ADRG vs. PRAD-25 | <0.001 |
| ADRG vs. PRAD-50 | <0.001 |
| PRAD-25 vs. PRAD-50 | <0.001 |
| F value | 1471.968 |
| df (df1 / df2) | 5 / 30 |
| *p* | <0.001 |

**Footnotes:** Intergroup differences were analyzed using one-way ANOVA, followed by Tukey’s Honestly Significant Difference (HSD) test for post hoc pairwise comparisons. For all groups, *n* = 6.

**Abbreviations**: PRO-25, propofol alone (25 mg/kg); PRO-50, propofol alone (50 mg/kg); ADRG, adrenaline alone (0.3 mg/kg); PRAD-25, adrenaline (0.3 mg/kg) combined with propofol (25 mg/kg); PRAD-50, adrenaline (0.3 mg/kg) combined with propofol (50 mg/kg); df, degrees of freedom.
